# Comparative transcriptomic analysis of thermally stressed *Arabidopsis thaliana* meiotic recombination mutants

**DOI:** 10.1186/s12864-021-07497-2

**Published:** 2021-03-12

**Authors:** Jiyue Huang, Hongkuan Wang, Yingxiang Wang, Gregory P. Copenhaver

**Affiliations:** 1 Department of Biology and the Integrative Program for Biological and Genome Sciences, University of North Carolina at Chapel Hill, Chapel Hill, North Carolina USA; 2 State Key Laboratory of Genetic Engineering and Collaborative Innovation Center of Genetics and Development, Ministry of Education Key Laboratory of Biodiversity Sciences and Ecological Engineering, Institute of Plant Biology, School of Life Sciences, Fudan University, Shanghai, China; 3 Lineberger Comprehensive Cancer Center, University of North Carolina School of Medicine, Chapel Hill, North Carolina USA

**Keywords:** Heat stress, Meiotic recombination, *MUS81*, *MSH4*, *ASY1*, RNA-Seq

## Abstract

**Background:**

Meiosis is a specialized cell division that underpins sexual reproduction in most eukaryotes. During meiosis, interhomolog meiotic recombination facilitates accurate chromosome segregation and generates genetic diversity by shuffling parental alleles in the gametes. The frequency of meiotic recombination in *Arabidopsis* has a U-shaped curve in response to environmental temperature, and is dependent on the Type I, crossover (CO) interference-sensitive pathway. The mechanisms that modulate recombination frequency in response to temperature are not yet known.

**Results:**

In this study, we compare the transcriptomes of thermally-stressed meiotic-stage anthers from *msh4* and *mus81* mutants that mediate the Type I and Type II meiotic recombination pathways, respectively. We show that heat stress reduces the number of expressed genes regardless of genotype. In addition, *msh4* mutants have a distinct gene expression pattern compared to *mus81* and wild type controls. Interestingly, *ASY1,* which encodes a HORMA domain protein that is a component of meiotic chromosome axes, is up-regulated in wild type and *mus81* but not in *msh4*. In addition, *SDS* the meiosis-specific cyclin-like gene, *DMC1* the meiosis-specific recombinase, *SYN1/REC8* the meiosis-specific cohesion complex component, and *SWI1* which functions in meiotic sister chromatid cohesion are up-regulated in all three genotypes. We also characterize 51 novel, previously unannotated transcripts, and show that their promoter regions are associated with A-rich meiotic recombination hotspot motifs.

**Conclusions:**

Our transcriptomic analysis of *msh4* and *mus81* mutants enhances our understanding of how the Type I and Type II meiotic CO pathway respond to environmental temperature stress and might provide a strategy to manipulate recombination levels in plants.

**Supplementary Information:**

The online version contains supplementary material available at 10.1186/s12864-021-07497-2.

## Background

Meiosis consists of a pair of cell divisions that produce gametes in sexually reproducing species. Prophase I of meiosis is distinct compared to mitosis because homologous chromosomes pair and reciprocally exchange DNA in a process called recombination. In most species, each pair of homologs must experience at least one exchange, or crossover (CO), in order to segregate properly at end of the first meiotic division. In the absence of a CO, chromosomes segregate randomly, resulting in chromosome number imbalances, or aneuploidy, in the gametes which, in turn, can result in developmental defects or lethality in progeny. Regulation of the frequency and distribution of COs in the genome has been studied extensively [1] and more recently, advances have been made in our understanding of how environmental conditions and stressors influence those regulatory mechanisms [2–4].

Numerous studies over the last century have demonstrated that environmental stressors, including thermal, nutrient, and drought stress can modulate CO frequencies in animals, fungi and plants [1, 5, 6]. The effect of temperature on meiotic recombination has been examined in several systems with results that suggest species-specific and complex regulatory mechanisms. In the model plant *Arabidopsis thaliana* for example, meiotic recombination exhibits a U-shaped response curve corresponding to variation from low to high environmental temperatures [3]. In other species, like *Hyacinthus orientalis* and *Oryza sativa*, meiotic recombination also increases with temperature, but in *Endymion nonscriptus* and *Rhoeo spathacea* it decreases [1, 5, 6]. In *Hordeum vulgare* (barley), a shift from 15 °C to 30 °C resulted in an incremental increase in male (but not female) CO frequencies, but also a distinct shift from proximal to distal COs along the chromosome arms [7]. At even higher temperatures, meiotic mechanisms begin to fail resulting in infertility [8, 9].

In plants, COs are divided into Type I, which are subject to a regulatory phenomenon known as crossover interference that inhibits closely spaced COs, and Type II which are insensitive to interference. MSH4 is a member of the ZMM (ZIP4, MSH4/5, MER3) group of proteins [10] that mediate the interference sensitive Type I pathway. *MSH4* encodes a meiosis-specific member of the MutS family of proteins responsible for DNA mismatch repair (MMR) in eukaryotes and prokaryotes. However, unlike other MutS family members, MSH4 does not show MMR activity. Instead, it is required for reciprocal meiotic recombination and proper homologous chromosome segregation [11]. MUS81*,* an endonuclease that resolves recombination intermediates, is required for Type II interference insensitive COs in most organisms [12]. In *Arabidopsis*, MSH4 mediates about 85% of COs, while MUS81 is responsible for 15% [11, 12]. Type I COs can also be quantified, and compared to genetic CO frequencies, using immunolocalization with antibodies to the MutL homolog, MLH3 [13]. Using this technique Phillips et al., showed that the high temperature-induced increase in the barley male genetic map was not accompanied by an increase in Type I COs [7], suggesting the hyper-recombinant response is instead mediated by the Type II pathway. These results stand in contrast to two recent studies in *Arabidopsis thaliana* that show a similar thermal-stress induced hyper-recombination phenotype, but use mutant analysis to demonstrate that the response is mediated by the Type I rather than Type II pathway [2, 3]. Thus, while the ability to modulate CO frequency in response to thermal stress is conserved between monocots and dicots, the specific pathways employed may differ significantly. Moreover, we know little about the gene products that sense environmental cues, and transduce them to the meiotic recombination machinery, although alteration of synaptonemal complex (SC) structure, modulation of chromatin states, and changes in the epigenetic landscape have been suggested as intriguing candidates [1, 5, 6].

To identify genes involved in mediating the thermal stress induced hyper-recombination phenotype, we performed a comparative analysis of the transcriptomes of meiotic stage anthers from wild type (WT; Col-0), *msh4* and *mus81* plants grown under control and thermally-stressed conditions. We found that heat stress reduces the number of gene expressed in all genotypes. WT and *mus81* plants have similar thermal-stress induced expression profiles which are distinct compared to *msh4*. Interestingly, the gene encoding the HORMA domain-containing chromosome axis protein *ASY1* is up-regulated in wild type and *mus81* but not in *msh4.* Additionally, we found 51 novel, unannotated transcripts that are associated with previously defined A-rich meiotic recombination hotspot motifs. Our transcriptomic analysis of meiotic Type I and Type II CO pathway mutants in temperature-stress conditions sheds new light on how abiotic factors regulate meiotic recombination in plants.

## Results

### mRNA-Seq characteristics of meiotic recombination pathway mutants

**Fig. 1:**
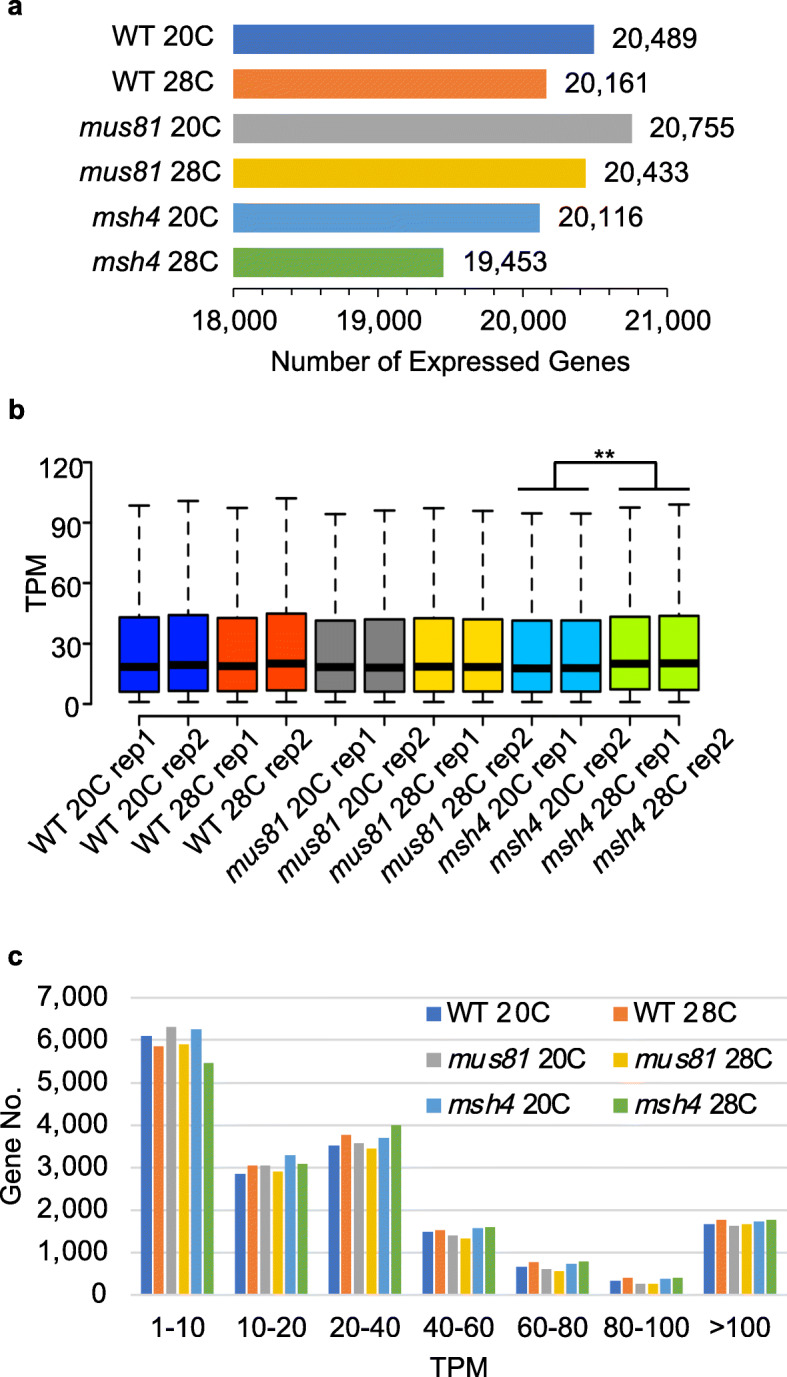
Characteristics of the heat-stressed and control transcriptomes in WT, *mus81* and *msh4*. **a**. The number of expressed genes in the six experimental samples. **b**. Gene expression value in all samples with two biological replicates (**P value < 2.2e-16 from Mann–Whitney test). **c**. Expressed genes grouped from low to high from all the six experimental samples

Previous reports from our lab and others revealed that the increased meiotic crossover frequencies observed in *Arabidopsis* grown at elevated temperatures are mediated by the Type I interference sensitive pathway [2, 3]. To identify genes potentially involved in the hyper-recombinant phenotype, we collected stage 4–7 anthers that contain male meiocytes from leptotene to tetrad stage [14] from WT (Col-0), *mus81*, and *msh4* plants. We collected two biological replicates for each genotype grown at 20 °C and 28 °C, then sequenced the 12 mRNA libraries. A total of 690,371,408 raw reads were retrieved from the 12 mRNA-Seq datasets with an average of 53 million mapped reads per dataset and a 93% average mapping rate (Supplementary Table 1). The biological replicates for each genotype-temperature combination had a minimum 0.92 correlation coefficient (Supplementary Table 2) indicating high reproducibility among our datasets. We examined the number of expressed genes in each condition with a threshold equal to or greater than 1 TPM (Transcripts Per Kilobase Million). At 20 °C, WT, *mus81* and *msh4* have 20,489, 20,755 and 20,116 expressed genes, respectively (Fig. 1a). After 28 °C heat stress treatment, the number of expressed genes decreased in all three genotypes (Fig. 1a). Both WT and *mus81* have 1.6% fewer expressed genes, while *msh4* has 3.3% fewer*.* We also compared the average level of gene expression in two biological replicates for each genotype at 20 °C and 28 °C. The TPM values do not differ significantly for WT (18.4 and 19.4 at 20 °C verus 18.8 and 20.1 at 28 °C, Mann–Whitney test, *P* value = 0.05) and *mus81* (18.4 and 18.1 at 20 °C verus 18.5 and 18.4 at 28 °C, Mann–Whitney test, *P* value = 0.18), but did for *msh4* (17.7 and 17.9 at 20 °C versus 20.0 and 20.2 at 28 °C; Mann–Whitney test, *P* value < 2.2e-16) (Fig. 1b), suggesting that heat stress decreases the number of expressed genes in *msh4* but increases the average strength of gene expression. We also compared the number of expressed genes in seven TPM value groups from low to high and found that all treatments and genotypes are similarly distributed, and that the group with TPM values between 1 and 10 is the largest (Fig. 1c). We then examined the overlap of expressed gene in WT, *mus81*, and *msh4* at 20 °C and 28 °C. At least 90% (18,398) of expressed genes are shared between all datasets, and 63 genes are shared by only WT and *mus81* at 28 °C (Supplementary Figure 1a). Only one enriched GO term, phospholipase C activity from Molecular Function (GO: 0004629, FDR = 0.02, by AT3G03530 and AT3G03540) was detected among these 63 genes, no meiotic recombination related genes were found, and most of the 63 genes have relatively low gene expression (Supplementary Figure 1b).

### WT and *mus81* have similar differential gene expression profiles

**Fig. 2:**
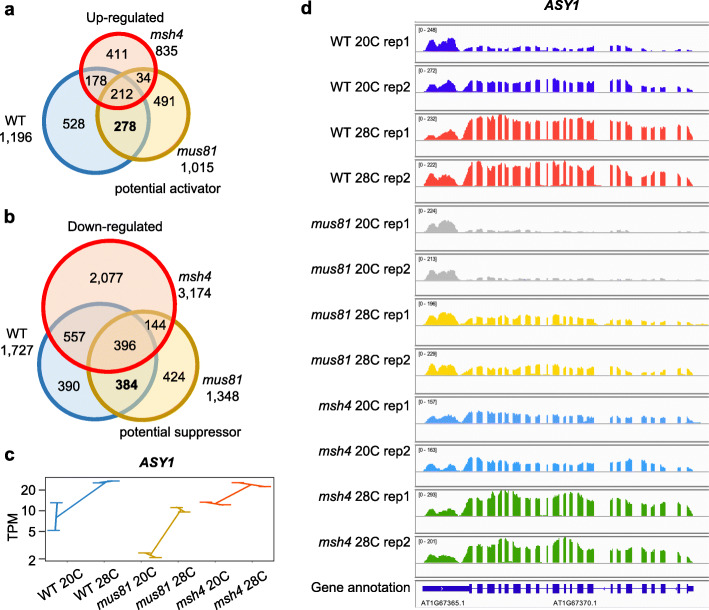
Characteristics of the differentially expressed genes (DEGs) from WT, *mus81* and *msh4 Arabidopsis* grown under 20 °C and 28 °C conditions. **a**. Venn diagram showing the common and specific up-regulated DEGs under heat stress among WT, *mus81* and *msh4*. **b**. Venn diagram showing the common and specific down-regulated DEGs under heat stress among WT, *mus81* and *msh4*. **c**. Expression of *ASY1* from WT, *mus81* and *msh4* grown under 20 °C and 28 °C conditions. **d**. Snapshot showing that *ASY1* is an up-regulated DEG in WT and *mus81* but not in *msh4*

By comparing expression levels of genes at 28 °C to those at 20 °C in each genotype, we found 2922, 2363 and 4009 differentially expressed genes (DEGs) from WT, *mus81* and *msh4*, respectively. Those include 1196 and 1727 up- and down-regulated genes in WT, 1015 and 1348 in *mus81*, and 835 and 3174 in *msh4*. We analyzed the intersections of the up- and down-regulated DEGs (Fig. 2a, b) to determine whether any of the genotypes had similar profiles. On a proportional basis, WT and *mus81* share 22% of their up-regulated DEGs and 25% of their down-regulated DEGs, WT and *msh4* share 19 and 19% respectively, and *mus81* and *msh4* share 13 and 12% respectively. WT and *mus81* share significantly more up-regulated DEGs compared to *mus81* and *msh4* (*χ*^*2*^ = 36.78, *P* value = 1.32E-09), and not significantly more to WT and *msh4* (*χ*^*2*^ = 3.56, *P* value = 0.06)*.* Similarly, WT and *mus81* share significantly more down-regulated DEGs compared to WT and *msh4* (*χ*^*2*^ = 24.51, *P* value = 7.38E-07), as well as *mus81* and *msh4* (*χ*^*2*^ = 158.02, *P* value < 2.2E-16)*.* It should be noted that disruption of the Type I CO pathway in *msh4* compromises but does not abolish pollen development and fertility, while WT and *mus81* are fertile, and that these differences in phenotype likely influence the differential gene expression patterns. Taken together, these data suggest that WT and *mus81* have the most similar DEG profiles of the genotypes examined (Fig. 2a).

**Fig. 3:**
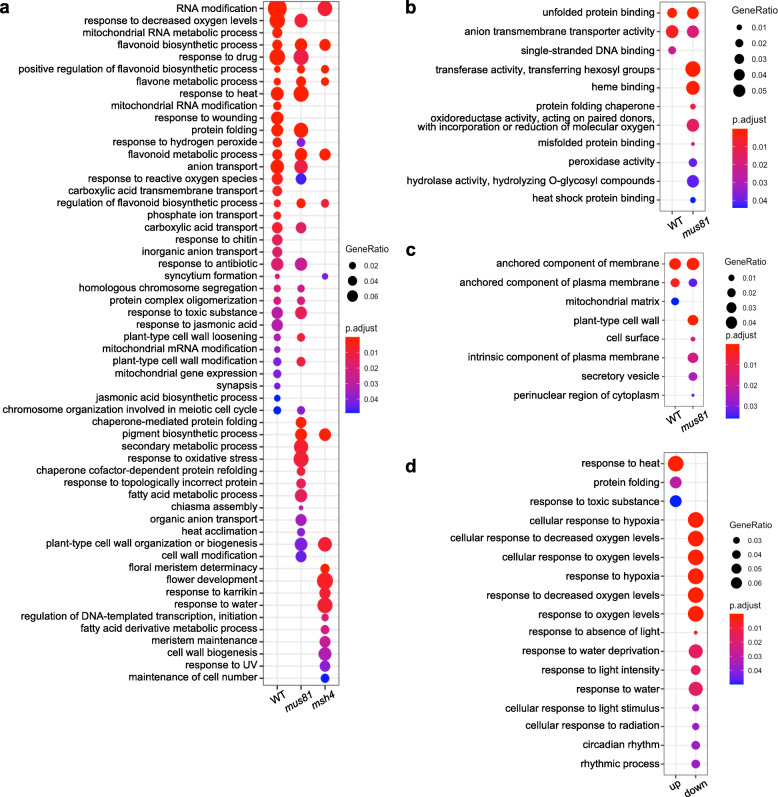
GO analysis of DEGs from WT, *mus81* and *msh4*. **a**. GO analysis of biological processes of up-regulated DEGs from WT, *mus81* and *msh4*. **b**. GO analysis of molecular functions of up-regulated DEGs from WT and *mus81.* Results in (**a** and **b**) were simplified by removing redundant GO terms, for full lists please see Supplementary Data 1. **c**. GO analysis of cellular components of up-regulated DEGs from WT and *mus81.* **d**. GO analysis of biological processes enriched in the 278 up-regulated DEGs shared by WT and *mus81*

We performed GO enrichment analysis to explore the characteristics of the DEGs from WT, *mus81* and *msh4* grown at 20 °C and 28 °C. Among the up-regulated DEGs, 45 Biological Process (BP), 7 Molecular Function (MF) and 3 Cellular Component (CC) GO terms were enriched in WT; 71, 19 and 7 in *mus81*; and 36, 0 and 0 in *msh4* (Supplementary Data 1). Several GO terms enriched among the 1196 DEGs from WT and 1015 from *mus81* are not enriched in the 835 DEGs from *msh4* including: 22 BP GO terms including cellular response to decreased oxygen levels (GO:0036294), response to heat (GO:0009408), protein folding (GO:0006457), homologous chromosome segregation (GO:0045143), and chromosome organization involved in meiotic cell cycle (GO:0070192), 4 MF GO terms including unfolded protein binding (GO:0051082) and 2 CC GO terms including anchored component of membrane (GO:0031225) (Fig. 3 and Supplementary Data 1). Among the down-regulated DEGs, there were 26 BP, 4 MF and 0 CC GO terms enriched in WT; 34, 18 and 8 in *mus81;* and 31, 13 and 9 in *msh4* (Supplementary Data 1). Only 6 BP GO terms, including response to absence of light (GO:0009646) and glucose import (GO:0046323), and 1 MF (carboxylic ester hydrolase activity; GO:0052689) are enriched in the down-regulated DEGs from WT and *mus81* (Supplementary Data 1 and Supplementary Figure 2). We noticed that *msh4* has the most down-regulated DEGs, and enriched GO terms not shared with WT or *mus81* that relate to male gametogenesis, such as pollen wall assembly (GO:0010208), pollen sperm cell differentiation (GO:0048235) and pollen exine formation (GO:0010584) (Supplementary Figure 2A). As mentioned above, this may be a consequence of the compromised pollen development phenotype observed in *msh4*.

**Fig. 4:**
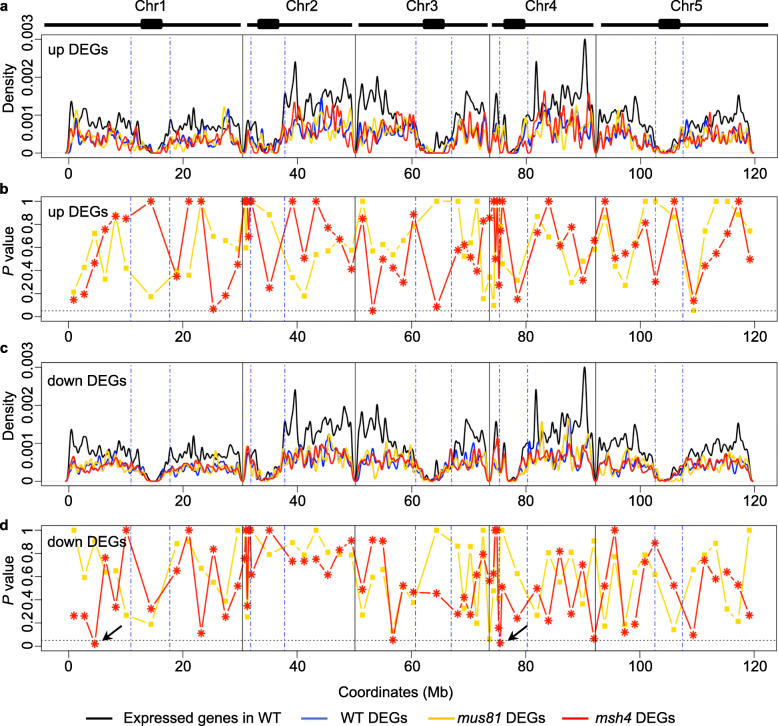
Genome-wide distribution of up- and down-regulated DEGs from WT, *mus81* and *msh4* along the *Arabidopsis* chromosomes. **a**. Density plot of up-regulated DEGs from WT, *mus81* and *msh4* along the *Arabidopsis* chromosomes with the distribution of expressed genes in WT as a control. **b**. Local enrichment analysis of up-regulated DEGs from *mus81* and *msh4.* **c**. Density plot of down-regulated DEGs from WT, *mus81* and *msh4* along the *Arabidopsis* chromosomes with the distribution of expressed genes in WT as a control. **d**. Local enrichment analysis of down-regulated DEGs from *mus81* and *msh4.* Chromosomes are partitioned into arm regions and centromeric regions (blue dashed lines) in (**a**, **b**, **c** and **d**). The horizontal black dashed lines in **b** and **d** indicate the *P* value = 0.05 from fisher test. Arrows in **d** show the two segments where there are significantly more down-regulated DEGs in *msh4* than in WT.

To determine whether the up- and down-regulated DEGs from WT, *mus81* and *msh4* display any pattern along the 5 *Arabidopsis* chromosomes we plotted their density as a function of physical position (Fig. 4a, c). We did not detect any global patterns in distribution: up-regulated DEGs from WT and *mus81* have a correlation coefficient of 0.46, while WT and *msh4* have a correlation coefficient of 0.42. Similarly, the down-regulated DEGs have a correlation coefficient of 0.51 between WT and *mus81* and 0.43 between WT and *msh4*. To examine the local distribution patterns of DEGs, we divided each chromosome into 12 euchromatic segments of equal length and one heterochromatic segment and tested whether the number of WT DEGs in each segment differs significantly from those of *mus81* or *msh4.* The number of up-regulated DEGs from *mus81* and *msh4* do not differ significantly from WT (Fig. 4b). Interestingly, two segments on the north arms of chromosome 1 and 4 show significantly more down-regulated DEGs in *msh4* than in WT (Fig. 4d). Overall, the distribution of DEGs does not differ dramatically among the genotypes but is consistent with WT and *mus81* being more similar to one another than *msh4.*

### GO terms enriched in the DEGs shared by WT and *mus81*

Exposure to elevated temperature results in an increase in Type I (MSH4-dependent) COs in *Arabidopsis* [2, 3]. To determine if that phenotype is correlated with changes in gene expression, we looked for DEGs shared between WT and *mus81* (in which the Type II pathway is inactive) but not *msh4*. We found 278 and 384 down-regulated DEGs that may be implicated in modulating the activity of the two pathways (Fig. 2a, b). GO analysis of the 278 up-regulated DEGs reveals enrichment of 3 BP GO terms: response to heat (GO:0009408), protein folding (GO:0006457) and response to toxic substance (GO:0009636), and 1 MF GO term: unfolded protein binding (GO:0051082) (Fig. 3d and Supplementary Data 2). Among the 384 down-regulated DEGs, 14 BP GO terms were enriched including: cellular response to decreased oxygen levels (GO:0036294), response to absence of light (GO:0009646), response to water deprivation (GO:0009414), and circadian rhythm (GO:0007623) (Fig. 3d and Supplementary Data 2).

### *ASY1* is exclusively up-regulated in WT and *mus81* but not in *msh4*

**Fig. 5:**
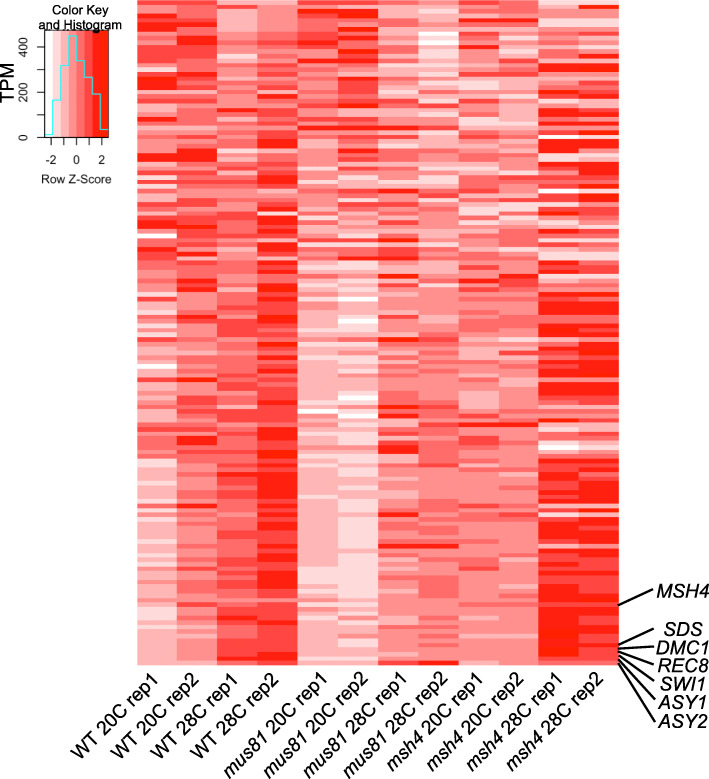
Heatmap of gene expression data from 148 gene with previously defined meiotic functions from WT, *mus81* and *msh4* plants grown at 20 °C and 28 °C. The ranges of colors in the heatmap represent lower (white) to higher (dark red) relative expression of each gene (rows) in each sample (columns)

We were interested in whether meiosis related genes are differentially expressed under heat stress. To this end, we examined the expression patterns of 148 genes that have been previously reported to have meiotic function (Supplementary Data 3). A heatmap comparison of these genes does not reveal any significant global difference in expression levels at 28 °C compared to 20 °C in WT, *mus81* or *msh4* (Fig. 5). To our surprise, *ASY1* and *ASY2* are the only consistently up-regulated DEGs exclusively shared by WT and *mus81* but not *msh4* (Fig. 2c, d; Supplementary Figure 3a, c) (*ASY1* and *ASY2* show an increase below the 2-fold cut-off at 28 °C compared to 20 °C in *msh4*, in contrast to 2.9 and 2.3-fold increases in WT, and 4.6 and 3.5-fold increases in *mus81*)*. ASY1* encodes a component of the chromosome axis that forms along the length of replicated sister chromatids during meiosis [15], and is required for proper interhomolog interaction including chromosome pairing, synapsis and recombination [16], as well as ensuring crossover interference [17]. *ASY2* is a putative functional homolog of *ASY1* [15]. No meiosis-related DEGs were found among the 384 down-regulated DEGs shared by WT and *mus81* (Supplementary Data 3). Interestingly, 4 genes are up-regulated in all three genotypes: the meiosis-specific cyclin-like gene *SDS* [18], meiosis-specific recombinase *DMC1* [19], meiosis-specific cohesion complex component *SYN1/REC8* [20]*,* and *SWI1* which functions in meiotic sister chromatid cohesion [21] (Supplementary Data 3). However, none of the 14 known meiotic genes involved in either the Type I or Type II CO pathways were differentially expressed (Table 1). The apparent up-regulation of *MSH4* in the *msh4* mutant background at 28 °C is most likely the result of the truncated *msh4* being driven by the 35S promoter carried by the T-DNA transgene insertion in this allele (Supplementary Figure 3B). Interestingly, we found one of the 21 *Arabidopsis* Heat Stress transcription Factor (HSF) genes, *AtHSFA2* (AT2G26150) [22], is among the 278 DEGs up-regulated in WT and *mus81* but not *msh4* (Supplementary Data 4). In addition, *AtHSFB2a* (AT5G62020) and *AtHSFA3* (AT5G03720) are up-regulated in WT, and *AtHSFB1* (AT4G36990) is up-regulated in *mus81*. Surprisingly, no HSF transcription factors are up-regulated in *msh4* (Supplementary Data 4). Further experimental work is needed to understand the potential interaction of the heat shock transcriptional regulatory networks and the meiotic recombination machinery.

**Table 1:** Expression value (TPM) of genes in meiotic recombination type I and type II pathways

Gene name	Alias	WT 20C rep1	WT 20C rep2	WT 28C rep1	WT 28C rep2	***mus81*** 20C rep1	***mus81*** 20C rep2	***mus81*** 28C rep1	***mus81*** 28C rep2	***msh4*** 20C rep1	***msh4*** 20C rep2	***msh4*** 28C rep1	***msh4*** 28C rep2
**Type I CO pathway regulators**													
**AT4G17380**	*MSH4*	7.3	9.8	9.0	10.4	5.3	4.6	5.8	7.4	8.6	9.6	38.0	50.1
**AT3G20475**	*MSH5*	20.4	25.2	20.4	24.5	14.6	12.3	19.0	20.3	23.3	20.5	16.5	16.5
**AT5G48390**	*ZIP4*	4.8	5.7	7.2	7.9	3.1	2.9	4.8	5.9	5.3	4.9	6.6	6.4
**AT3G27730**	*RCK/MER3*	4.8	8.1	6.3	8.2	3.0	2.7	4.6	5.1	7.6	5.5	8.0	7.8
**AT1G12790**	*PTD*	15.1	15.4	14.5	17.2	8.9	12.3	9.9	11.8	14.3	9.7	12.9	13.4
**AT5G52290**	*SHOC1/ZIP2*	1.3	2.0	2.5	3.4	1.0	1.0	1.2	1.6	1.8	1.6	3.1	3.1
**AT1G53490**	*HEI10*	6.9	9.2	9.7	10.7	26.5	28.1	27.6	22.9	7.4	6.3	12.6	11.5
**AT4G09140**	*MLH1*	26.5	26.8	26.3	29.6	21.2	21.0	23.0	21.5	24.8	26.4	27.2	26.2
**AT4G35520**	*MLH3*	6.8	7.5	6.8	9.1	5.8	5.9	9.0	6.8	7.1	8.1	4.8	5.9
**AT1G63770**	*MPA1*	122.1	115.0	116.4	128.4	105.0	96.5	111.5	114.4	117.6	120.3	124.8	119.7
**Type II CO pathway regulators**													
**AT4G30870**	*MUS81*	22.6	24.0	25.0	30.2	4.5	5.4	5.0	4.9	21.0	20.2	17.2	19.1
**AT2G21800**	*EME1A*	4.4	5.6	7.5	7.2	4.7	5.7	7.0	5.6	4.7	5.5	8.4	9.2
**AT2G22140**	*EME1B*	3.1	4.2	6.0	5.6	3.8	3.0	2.9	3.2	3.7	3.4	5.8	5.4
**AT4G14970**	*FANCD2*	5.9	8.3	10.7	12.1	5.2	4.6	7.1	7.9	7.8	7.6	12.9	14.1

### Novel anther-specific transcripts associated with A-rich promoter motifs

**Fig. 6:**
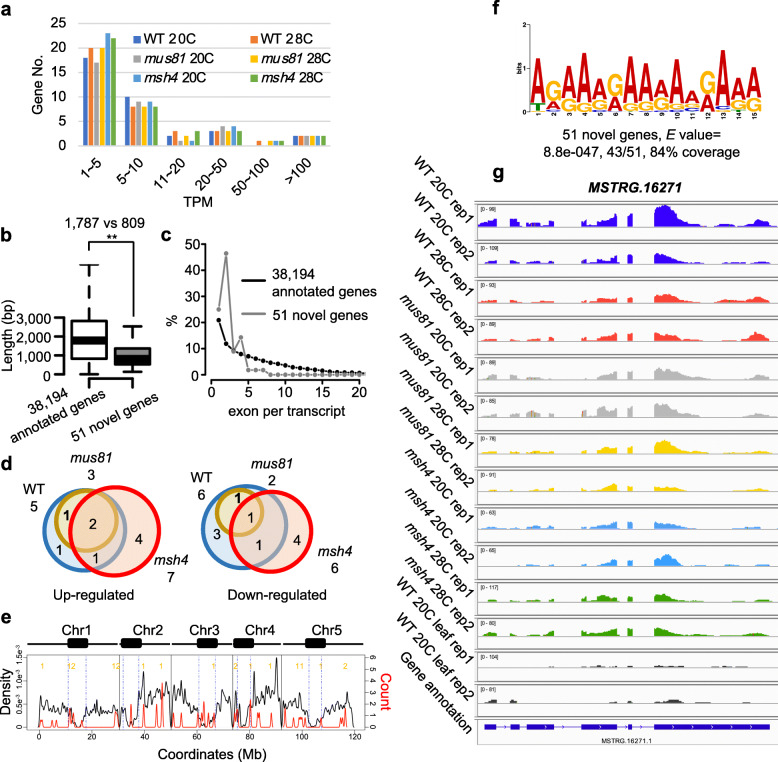
Characteristics of novel transcripts from anther derived samples. **a** Novel transcripts grouped from low to high from all the six experimental samples. **b** Comparison of transcript length from 51 novel transcripts and 38,194 annotated genes (**P value < 2.2e-16 from Mann–Whitney test). **c** Comparison of exon number per transcript from 51 novel transcripts and 38,194 annotated genes. **d** Venn diagram showing the common and specific differential expressed novel transcripts under heat stress among WT, *mus81* and *msh4*. **e** Density plot of 51 novel transcripts along the *Arabidopsis* chromosomes with the distribution of expressed genes in WT as a control. Numbers in yellow indicate the positions of the 19 anther-specific novel transcripts (1 novel transcript localizes in the Mitochondrial DNA). **f** An A-rich motif is enriched in putative promoter sequences of the 51 novel transcripts. **g** Snapshot showing the anther-specific novel transcript *MSTRG.16271*

Our RNA-seq analysis of anthers identified 51 novel transcripts that had not been previously annotated in the *Arabidopsis* genome (Supplementary Data 5). 39% of the novel transcripts have relatively low TPM values between 1 and 5, 14% have TPMs of 5–10 (Fig. 6a) similar to previously annotated genes, 35% of which have TPMs of 1–10 (Fig. 1c). The novel transcripts are short with a median length of 809 bp compared to 1787 bp from the 38,194 annotated genes (Fig. 6b). However, 26 (46%) of the novel transcripts have 2 exons, 14 (25%) have 1, and 8 (14%) have 4 exons, compared to annotated genes in which the most abundant group (21%) has only one exon (Fig. 6c). Comparison of the transcripts expressed at 20 °C and 28 °C in the different genotypes reveals Differentially Expressed Novel Transcripts (DENTs) which include 5, 3 and 7 up-regulated and 6, 2 and 6 down-regulated DENTs from WT, *mus81* and *msh4,* respectively (Fig. 6d). Noticeably, all DENTs in *mus81* are shared by WT (Fig. 6d), which is consistent with the previous observations that the transcriptome profiles of WT and *mus81* are more similar compared to *msh4*.

The loci encoding the novel transcripts are not preferentially partitioned into euchromatin or heterochromatin (Fisher’s Exact Test, *P* value = 0.18) (Fig. 6e). However, we wondered whether they are expressed in a tissue-specific manner. To address this question, we generated two RNA-seq datasets from WT leaf tissue and compared them to the anther datasets (Supplementary Table 1). We found that 20 (40%) of the 51 novel transcripts are anther-specific and 5 more are preferentially expressed in the anther when comparing 20 °C anther datasets to WT leaves (Fig. 6e; Supplementary Data 5). Like the larger novel transcript set, the 20 anther-specific transcripts are not preferentially partitioned into euchromatin or heterochromatin (Fisher’s Exact Test, *P* value = 0.29) (Fig. 6e). To test whether these anther-specific novel transcripts are regulated by similar cis-regulatory elements we used MEME to search for conserved DNA motifs [23]. 43 (84%) of the novel transcripts had an upstream A-rich motif in their putative promoter regions, including 90% (18 from 20) of the anther-specific novel transcripts (*E* value = 8.8e-047) (Fig. 6f). These A-rich motifs have been reported to be associated with open chromatin structure and meiotic recombination hotspots [24–27], suggesting that the anther-specific novel transcripts may be expressed in response to a change in chromatin state associated with meiotic recombination. Moreover, of the 51 novel transcripts we identified 9 can potentially code for proteins (Supplementary Data 6). Of those *MSTRG.16271*, resembles a *Danio rerio* gene that encodes the guanine nucleotide exchange factor subunit RIC1 (Fig. 6g).

## Discussion

Meiotic recombination is genetically regulated but also responds to external factors like temperature, drought and nutritient availability [5, 6]. However, the underlining molecular mechanisms that facilitate the regulation of meiotic recombination are still largely uncharacterized. Previous studies from our lab and others have shown that moderate heat stress results in an increase in Type I COs in *Arabidopsis* [2, 3]. Here we analyzed the transcriptomes of Type I and Type II CO pathways mutants *msh4* and *mus81*, under normal and heat stress growth conditions, and found that fewer genes are expressed after heat stress regardless of genotype (Fig. 1a). Other meiotic changes have also been observed in response to heat stress including a negative correlation between *Arabidopsis* synaptonemal complex (SC) length and temperature [3]. The Type I pathway mutant *msh4* has the fewest expressed genes at 20 °C, and 3.3% fewer at 28 °C (Fig. 1a). Consistent with this observation, we identified more down-regulated DEGs in *msh4* as compared to WT or *mus81* (Fig. 2b). It will be interesting to know whether the transcriptional changes we have documented are directly connected, on a mechanistic level, with regulating SC length or CO number.

We observed that *ASY1* and *ASY2* are the only previously characterized meiotic recombination-associated genes that are significantly up-regulated in WT and *mus81* but not in *msh4* (Fig. 2). This is consistent with previous observations that barley *ASY1* is prematurely up-regulated in early stage anthers under high-temperature conditions [28]. MUS81 mediates the CO interference insensitive (Type II) meiotic pathway, while *msh4* is required for the interference sensitive (Type I) pathway. This suggests that *ASY1* and *ASY2* may potentially function in tuning the balance between meiotic recombination pathways in response to external cues like heat stress. In *Arabidopsis,* ASY1 is known to antagonize telomere-led recombination in a dosage-dependent manner [17]. In autotetraploid *Arabidopsis arenosa*, different *ASY1* alleles influence meiotic chromosome interactions, morphology and axis length [29], and *ASY1* is among the key axis component genes that are under strong selection [30]. These results are consistent with the hypothesis that the regulation of *ASY1* gene expression influences meiotic recombination levels. However, recent studies in *Arabidopsis* reported no change in ASY1 immunostaining in wild type meiocytes after 30–32 °C heat shock for 24 h [4], but partially compromised localization under 36–38 °C extreme heat stress conditions [31]. It is important to note that the regulatory responses to moderate environmental stresses may not be reflected in more extreme conditions which can instead result in meiotic failure.

We identified 51 novel transcripts from our anther derived RNA-seq datasets (Fig. 6) including 20 that are anther-specific (Fig. 6) and 9 that have open reading frames encoding more than 100 amino acids (Supplementary Data 6). *Arabidopsis thaliana* has a mature and well annotated genome but the latest Arabidopsis annotation (Araport11) was constructed from 11 tissues that did not specifically include early stage floral buds that contain anthers undergoing meiosis (though later stage anthers were included) [32]. Similarly, the libraries used for the annotation did not include plants that had been subjected to abiotic stress conditions. Thus, it is not unreasonable that we identified several previously unannotated transcripts since we were examing an under-represented tissue type in specific environmental conditions. Interestingly, we found that the upstream putative promoter regions of the loci encoding the 51 novel transcripts are enriched for a known meiotic recombination hotspot-associated A-rich motif (Fig. 6f). The relevance of this finding may be somewhat tempered by the fact that A-rich elements are abundant in the genome and are also significantly enriched in the set of all known gene promoters (*E* value = 5.3e-103) (Supplementary Figure 4). At the very least, the association of the A-rich motif with these 51 novel transcript loci suggests that they are bona fide genes. More speculatively, since the A-rich motif has been reported to be associated with open chromatin structure and meiotic recombination hotspots [24–27], it is possible that these novel transcripts are up-regulated as the result of changes in chromatin structure associated with meiotic recombination.

## Conclusions

By comparing thetranscriptional profiles of *Arabidopsis* wild type and meiotic recombination mutants under normal and heats-stress conditions, we show that mutants in the Type II meiotic recombination pathway (*mus81*) are more similar to WT than mutants in the Type I pathway (*msh4*). The chromosomal axis protein ASY1 is up-regulated in both WT and *mus81*, but not *msh4*. We also describe 51 novel transcripts that are expressed in meiotic tissues, including several that are differentially expressed under heat stress conditions. Interestingly, the loci encoding these novel transcripts are enriched for a previously defined A-rich DNA motif that have been associated with open chromatin and meiotic recombination hotspots. We believe this work will be of interest to the meiosis community.

## Methods

### Plant materials and growth conditions

*Arabidopsis thaliana* lines including wild type Col-0, *mus81* (SALK_107515) and *msh4* (SALK_136296) used in this study were obtained from the Arabidopsis Biological Resource Center. Plants were grown in soil under long day (16 h light and 8 h dark) growth room conditions at 20 °C. For the heat treatment, flowering plants were placed in a 28 °C growth chamber for 5 days. Anthers at stage 4–7 [14] were collected using a dissecting microscope on the fifth day, frozen in liquid nitrogen and stored in − 80 °C for later use. Genotyping primers are provided in Supplementary Table 3.

### Transcriptome sequencing, data collection, and analysis

Total RNA was extracted using Trizol (Thermo Fisher Scientific) following the manufacturer’s protocol from stage 4–7 anthers collected as described above. mRNA-Seq libraries were constructed using TruSeq RNA Library Preparation Kits (Illumina). Paired-end sequencing was performed with an Illumina Hiseq 3000. Retrieved raw reads were trimmed using BBMap (version 38.82, Bushnell B., https://sourceforge.net/projects/bbmap/) to remove adapters. Whole genome sequence and Araport11 annotation were downloaded from TAIR10 (https://www.Arabidopsis.org/). Filtered reads were mapped using TopHat2 [33] then analyzed using Stringtie [34] and DESeq2 [35]. TPM values of each gene from all conditions were calculated using Stringtie with the parameter “-A” [34]. An expressed gene was defined using a cut off of ≥1 TPM in both biological replicates. Differentially expressed genes were analysed using DESeq2 using the following criteria: log2fdc > 1 or < − 1, q value < 0.05. GO analyses were performed in R using the clusterProfiler package [36]. GO term enrichment plot results were simplified with the command “simplify” (x, cutoff = 0.7, by = “p.adjust”, select_fun = min). Illustrations of gene expression profiles were plotted using the Integrative Genomics Viewer (IGV 2.8.2). Pearson’s product-moment correlation tests were performed in R. Chromosome local DEGs enrichment tests were calculated using fisher test in R.

### DNA motif discovery

We defined the 1000 bp sequence upstream of each novel transcript as its putative promoter. Potential cis-regulatory element or conserved DNA motif discovery of the 51 putative promoter sequences was analyzed using MEME (5.1.1) [23] with the default parameters with the following exceptions: -mod anr -minw 5 -maxw 30 -revcomp.

### Novel transcript analysis

Novel transcripts were discovered using Stringtie with a parameter “-m 30” [34]. We used a cut off of ≥1 TPM in both biological replicates in the WT 20C, WT 28C, *mus81* 20C, *mus81* 28C, *msh4* 20C or *msh4* 28C datasets to define the novel transcripts. Anther-specific novel transcripts were defined as those that had ≥1 TPM in both anther datasets, but < 1 TPM in both biological replicates from the leaves. Novel preferentially expressed anther transcripts were defined as those that had > 1 TPM in both biological replicates in leaves and significantly higher TPM values in WT 20C, mus81 20C and, msh4 20C compared to leaves using DESeq2 [35], log2fdc > 1, q value < 0.05. Candidate coding regions within transcript sequences and potential protein sequences were identified by using TransDecoder with its default setting (https://github.com/TransDecoder/TransDecoder/). Only protein sequences larger than 100 amino acid were retained.

## Supplementary Information

**Additional file 1 **: **Supplementary Table 1.** Characteristics of the 14 RNA-Seq datasets used in the study.

**Additional file 2 **: **Supplementary Table 2.** Pearson’s product moment correlation coefficients (r) between pairs of samples used in this study.

**Additional file 3 **: **Supplementary Table 3.** Genotyping primers used in this study.

**Additional file 4 **: **Supplementary Figure 1.** Intersection of expressed genes among all 6 samples in **a**, and expressed gene numbers grouped from low to high of the 63 genes only expressed in WT and *mus81* at 28 °C in **b**.

**Additional file 5 **: **Supplementary Figure 2.** GO analysis of biological processes **a**, molecular functions **b** and cellular components **c** of down-regulated DEGs from WT, *mus81* and *msh4*.

**Additional file 6 **: **Supplementary Figure 3.** Gene expression of *ASY2* and *MSH4* under heat stress in WT, *mus81* and *msh4*. **a** Snapshot showing that *ASY2* is an up-regulated DEG in WT and *mus81* but not in *msh4*. **b** Snapshot showing atypical up-regulated *MSH4* in *msh4* but not in WT and *mus81*. **c** Expression of *ASY2* from WT, *mus81* and *msh4* grown under 20 °C and 28 °C conditions.

**Additional file 7 **: **Supplementary Figure 4.** A-rich motif enriched in the promoter sequences of 51 randomly selected genes from *Arabidopsis* Araport11 annotation set.

**Additional file 8 **: **Supplementary Data 1.** Enriched GO terms from up- and down-regulated DEGs in WT, *mus81* and *msh4* after heat stress.

**Additional file 9 **: **Supplementary Data 2.** Enriched GO terms from 278 up- and 384 down-regulated DEGs found in WT and *mus81* after heat stress but not in *msh4*.

**Additional file 10 **: **Supplementary Data 3.** Expression data from 148 genes with previously described meiotic functions in WT, *mus81* and *msh4* at 20 °C and 28 °C.

**Additional file 11 **: **Supplementary Data 4.** Expression data from the 21 Arabidopsis HSF genes in WT, *mus81* and *msh4* at 20 °C and 28 °C.

**Additional file 12 **: **Supplementary Data 5.** 51 novel transcripts described in this study.

**Additional file 13 **: **Supplementary Data 6.** Potential Open Reading Frames (ORFs) of the 9 novel genes.

## Data Availability

The datasets supporting the conclusions of this article are available in the NCBI Sequence Read Archive (SRA) repository under the project name PRJNA679774 (https://www.ncbi.nlm.nih.gov/bioproject/ PRJNA679774). All other datasets generated for this study are included in the article/Supplementary Material, further inquiries can be directed to the corresponding authors.

## Abbreviations

BP: Biological process
CC: Cellular component
CO: Crossover
DEG: Differential expressed gene
DENT: Differentially Expressed Novel Transcript
HSF: Heat stress transcription factor
MF: Molecular function
MMR: Mismatch repair
SC: Synaptonemal complex
TPM: Transcripts Per kilobase Million
WT: Wildtype
