# Supplementary material for: Comparative transcriptomic analysis of thermally stressed Arabidopsis thaliana meiotic recombination mutants
Source: BMC Genomics. 2021 Mar 12;22:181. doi: 10.1186/s12864-021-07497-2 (PMC7953577; doi:10.1186/s12864-021-07497-2)
Supplement: Supplementary file 1 — Additional file 1 : Supplementary Table 1. Characteristics of the 14 RNA-Seq datasets used in the study. [file 12864_2021_7497_MOESM1_ESM.pptx]

## Slide 1
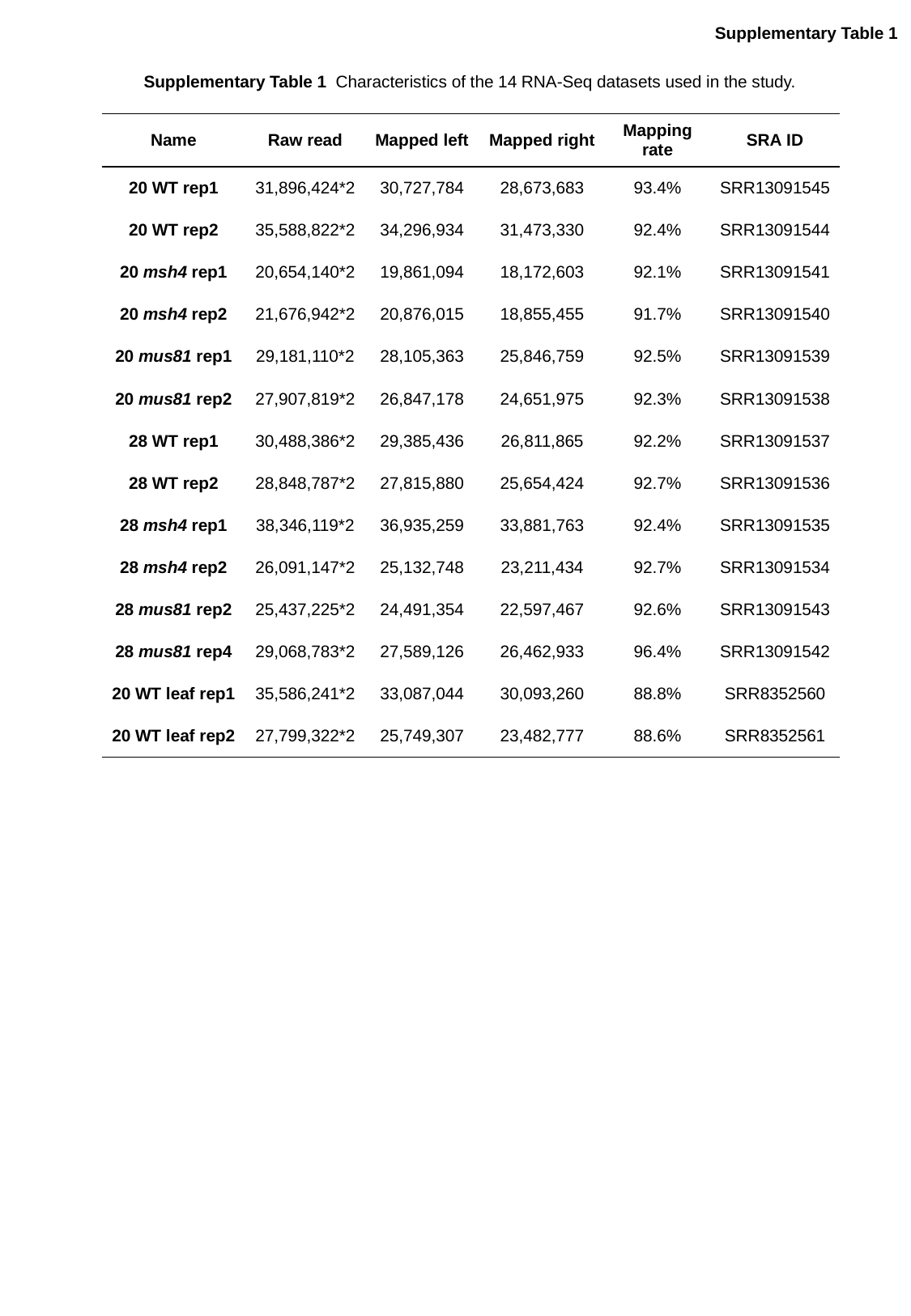

Supplementary Table 1
Supplementary Table 1 Characteristics of the 14 RNA-Seq datasets used in the study.
| Name | Raw read | Mapped left | Mapped right | Mapping rate | SRA ID |
| --- | --- | --- | --- | --- | --- |
| 20 WT rep1 | 31,896,424\*2 | 30,727,784 | 28,673,683 | 93.4% | SRR13091545 |
| 20 WT rep2 | 35,588,822\*2 | 34,296,934 | 31,473,330 | 92.4% | SRR13091544 |
| 20 msh4 rep1 | 20,654,140\*2 | 19,861,094 | 18,172,603 | 92.1% | SRR13091541 |
| 20 msh4 rep2 | 21,676,942\*2 | 20,876,015 | 18,855,455 | 91.7% | SRR13091540 |
| 20 mus81 rep1 | 29,181,110\*2 | 28,105,363 | 25,846,759 | 92.5% | SRR13091539 |
| 20 mus81 rep2 | 27,907,819\*2 | 26,847,178 | 24,651,975 | 92.3% | SRR13091538 |
| 28 WT rep1 | 30,488,386\*2 | 29,385,436 | 26,811,865 | 92.2% | SRR13091537 |
| 28 WT rep2 | 28,848,787\*2 | 27,815,880 | 25,654,424 | 92.7% | SRR13091536 |
| 28 msh4 rep1 | 38,346,119\*2 | 36,935,259 | 33,881,763 | 92.4% | SRR13091535 |
| 28 msh4 rep2 | 26,091,147\*2 | 25,132,748 | 23,211,434 | 92.7% | SRR13091534 |
| 28 mus81 rep2 | 25,437,225\*2 | 24,491,354 | 22,597,467 | 92.6% | SRR13091543 |
| 28 mus81 rep4 | 29,068,783\*2 | 27,589,126 | 26,462,933 | 96.4% | SRR13091542 |
| 20 WT leaf rep1 | 35,586,241\*2 | 33,087,044 | 30,093,260 | 88.8% | SRR8352560 |
| 20 WT leaf rep2 | 27,799,322\*2 | 25,749,307 | 23,482,777 | 88.6% | SRR8352561 |
