# Supplementary material for: Comparative transcriptomic analysis of thermally stressed Arabidopsis thaliana meiotic recombination mutants
Source: BMC Genomics. 2021 Mar 12;22:181. doi: 10.1186/s12864-021-07497-2 (PMC7953577; doi:10.1186/s12864-021-07497-2)
Supplement: Supplementary file 2 — Additional file 2 : Supplementary Table 2. Pearson’s product moment correlation coefficients (r) between pairs of samples used in this study. [file 12864_2021_7497_MOESM2_ESM.pptx]

## Slide 1
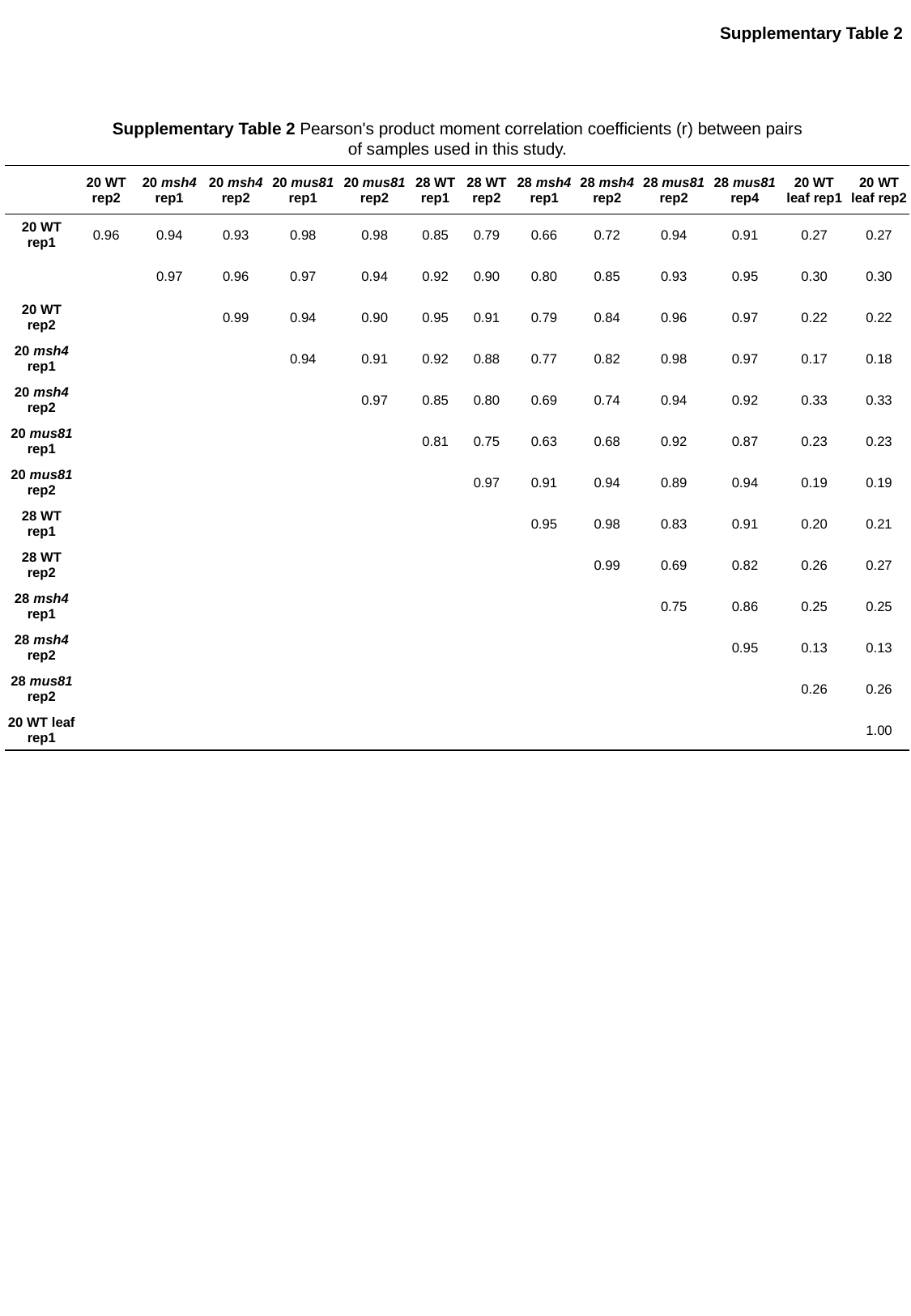

Supplementary Table 2
Supplementary Table 2 Pearson's product moment correlation coefficients (r) between pairs of samples used in this study.
| | 20 WT rep2 | 20 msh4 rep1 | 20 msh4 rep2 | 20 mus81 rep1 | 20 mus81 rep2 | 28 WT rep1 | 28 WT rep2 | 28 msh4 rep1 | 28 msh4 rep2 | 28 mus81 rep2 | 28 mus81 rep4 | 20 WT leaf rep1 | 20 WT leaf rep2 |
| --- | --- | --- | --- | --- | --- | --- | --- | --- | --- | --- | --- | --- | --- |
| 20 WT rep1 | 0.96 | 0.94 | 0.93 | 0.98 | 0.98 | 0.85 | 0.79 | 0.66 | 0.72 | 0.94 | 0.91 | 0.27 | 0.27 |
| | | 0.97 | 0.96 | 0.97 | 0.94 | 0.92 | 0.90 | 0.80 | 0.85 | 0.93 | 0.95 | 0.30 | 0.30 |
| 20 WT rep2 | | | 0.99 | 0.94 | 0.90 | 0.95 | 0.91 | 0.79 | 0.84 | 0.96 | 0.97 | 0.22 | 0.22 |
| 20 msh4 rep1 | | | | 0.94 | 0.91 | 0.92 | 0.88 | 0.77 | 0.82 | 0.98 | 0.97 | 0.17 | 0.18 |
| 20 msh4 rep2 | | | | | 0.97 | 0.85 | 0.80 | 0.69 | 0.74 | 0.94 | 0.92 | 0.33 | 0.33 |
| 20 mus81 rep1 | | | | | | 0.81 | 0.75 | 0.63 | 0.68 | 0.92 | 0.87 | 0.23 | 0.23 |
| 20 mus81 rep2 | | | | | | | 0.97 | 0.91 | 0.94 | 0.89 | 0.94 | 0.19 | 0.19 |
| 28 WT rep1 | | | | | | | | 0.95 | 0.98 | 0.83 | 0.91 | 0.20 | 0.21 |
| 28 WT rep2 | | | | | | | | | 0.99 | 0.69 | 0.82 | 0.26 | 0.27 |
| 28 msh4 rep1 | | | | | | | | | | 0.75 | 0.86 | 0.25 | 0.25 |
| 28 msh4 rep2 | | | | | | | | | | | 0.95 | 0.13 | 0.13 |
| 28 mus81 rep2 | | | | | | | | | | | | 0.26 | 0.26 |
| 20 WT leaf rep1 | | | | | | | | | | | | | 1.00 |
