# Supplementary material for: Comparative transcriptomic analysis of thermally stressed Arabidopsis thaliana meiotic recombination mutants
Source: BMC Genomics. 2021 Mar 12;22:181. doi: 10.1186/s12864-021-07497-2 (PMC7953577; doi:10.1186/s12864-021-07497-2)
Supplement: Supplementary file 3 — Additional file 3 : Supplementary Table 3. Genotyping primers used in this study. [file 12864_2021_7497_MOESM3_ESM.pptx]

## Slide 1
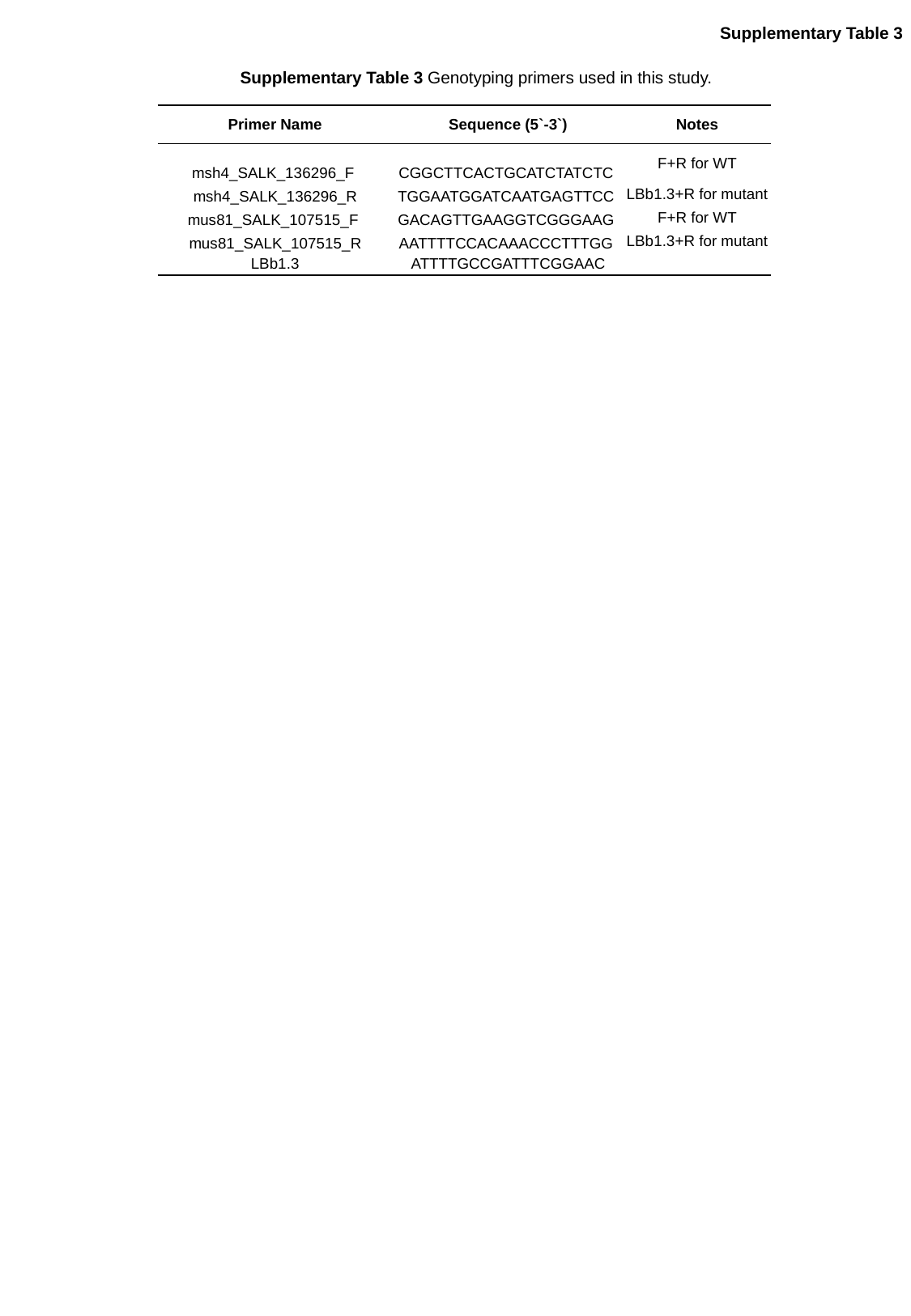

Supplementary Table 3
Supplementary Table 3 Genotyping primers used in this study.
| Primer Name | Sequence (5`-3`) | Notes |
| --- | --- | --- |
| msh4\_SALK\_136296\_F | CGGCTTCACTGCATCTATCTC | F+R for WT |
| msh4\_SALK\_136296\_R | TGGAATGGATCAATGAGTTCC | LBb1.3+R for mutant |
| mus81\_SALK\_107515\_F | GACAGTTGAAGGTCGGGAAG | F+R for WT |
| mus81\_SALK\_107515\_R | AATTTTCCACAAACCCTTTGG | LBb1.3+R for mutant |
| LBb1.3 | ATTTTGCCGATTTCGGAAC | |
