# Supplementary material for: Comparative transcriptomic analysis of thermally stressed Arabidopsis thaliana meiotic recombination mutants
Source: BMC Genomics. 2021 Mar 12;22:181. doi: 10.1186/s12864-021-07497-2 (PMC7953577; doi:10.1186/s12864-021-07497-2)
Supplement: Supplementary file 4 — Additional file 4 : Supplementary Figure 1. Intersection of expressed genes among all 6 samples in a, and expressed gene numbers grouped from low to high of the 63 genes only expressed in WT and mus81 at 28 °C in b. [file 12864_2021_7497_MOESM4_ESM.pdf]

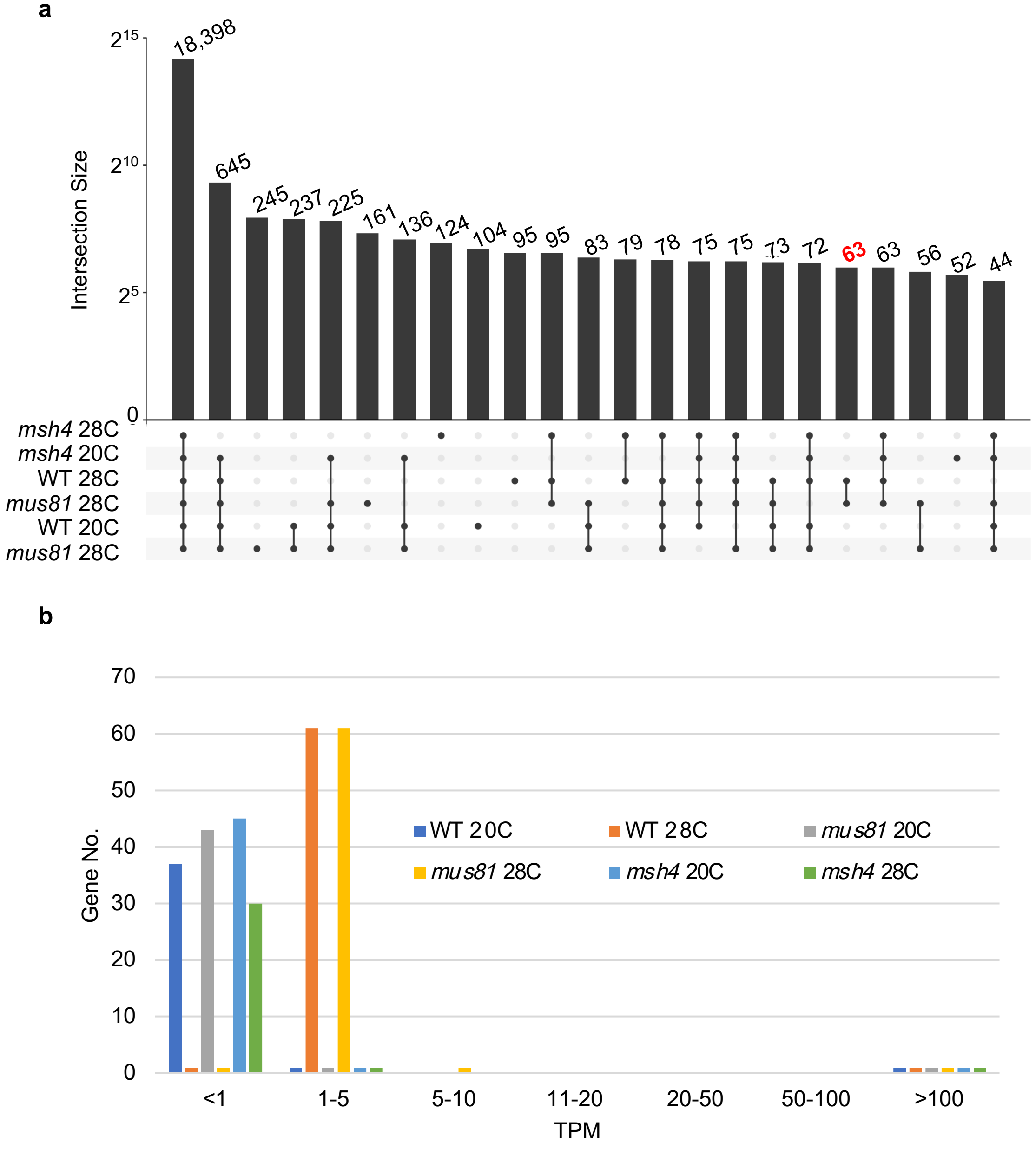


**Supplementary Figure 1** Intersection of expressed genes among all 6 samples in **a**, and expressed gene numbers grouped from low to high of the 63 genes only expressed in WT and *mus81* at 28°C in **b**.
