# Supplementary material for: Comparative transcriptomic analysis of thermally stressed Arabidopsis thaliana meiotic recombination mutants
Source: BMC Genomics. 2021 Mar 12;22:181. doi: 10.1186/s12864-021-07497-2 (PMC7953577; doi:10.1186/s12864-021-07497-2)
Supplement: Supplementary file 5 — Additional file 5 : Supplementary Figure 2. GO analysis of biological processes a, molecular functions b and cellular components c of down-regulated DEGs from WT, mus81 and msh4. [file 12864_2021_7497_MOESM5_ESM.pdf]

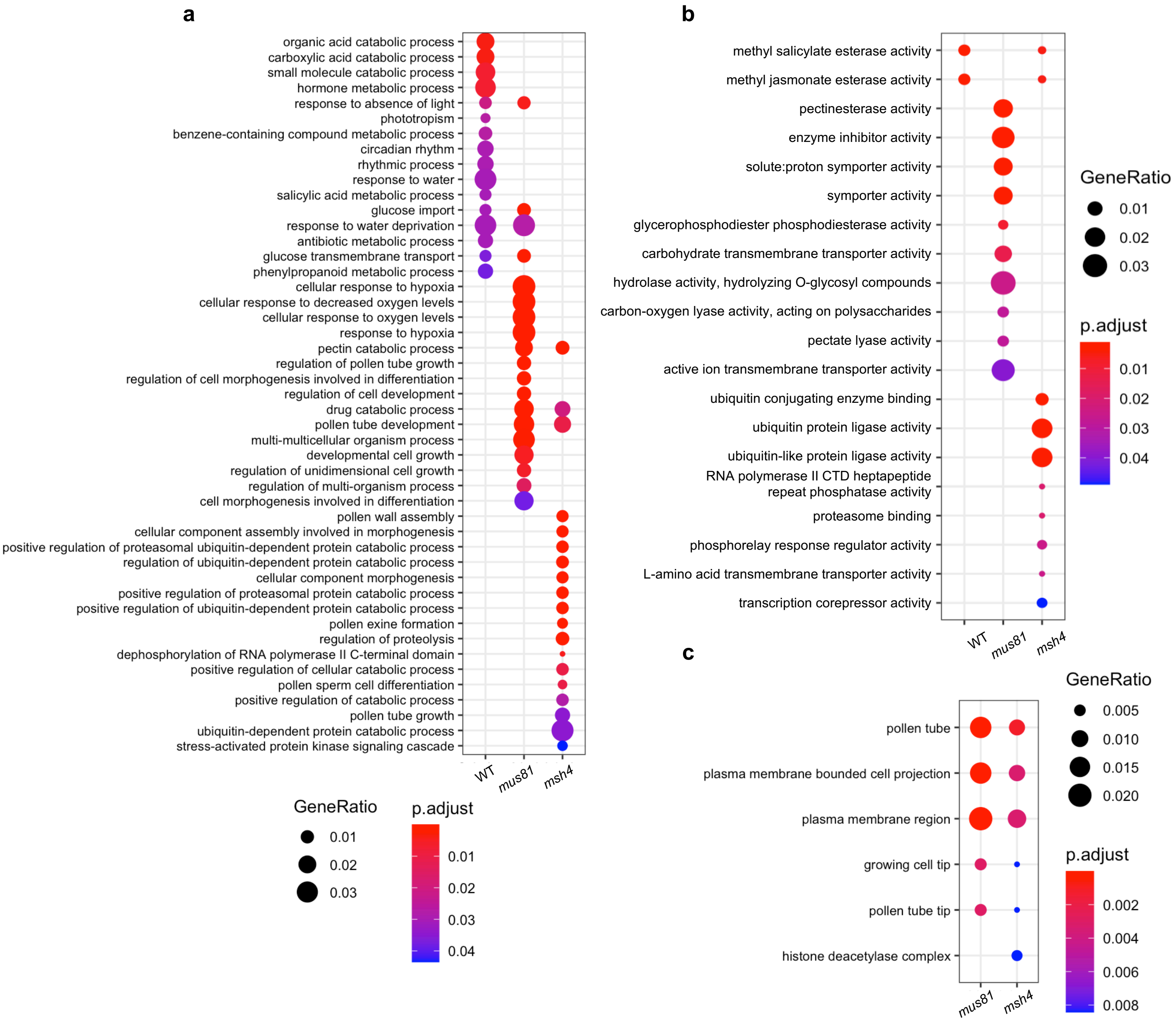

Supplementary Figure 2 GO analysis of biological processes **a**, molecular functions **b** and cellular components **c** of down-regulated DEGs from WT, *mus81* and *msh4*.
