# Supplementary material for: Comparative transcriptomic analysis of thermally stressed Arabidopsis thaliana meiotic recombination mutants
Source: BMC Genomics. 2021 Mar 12;22:181. doi: 10.1186/s12864-021-07497-2 (PMC7953577; doi:10.1186/s12864-021-07497-2)
Supplement: Supplementary file 6 — Additional file 6 : Supplementary Figure 3. Gene expression of ASY2 and MSH4 under heat stress in WT, mus81 and msh4. a Snapshot showing that ASY2 is an up-regulated DEG in WT and mus81 but not in msh4. b Snapshot showing atypical up-regulated MSH4 in msh4 but not in WT and mus81. c Expression of ASY2 from WT, mus81 and msh4 grown under 20 °C and 28 °C conditions. [file 12864_2021_7497_MOESM6_ESM.pdf]

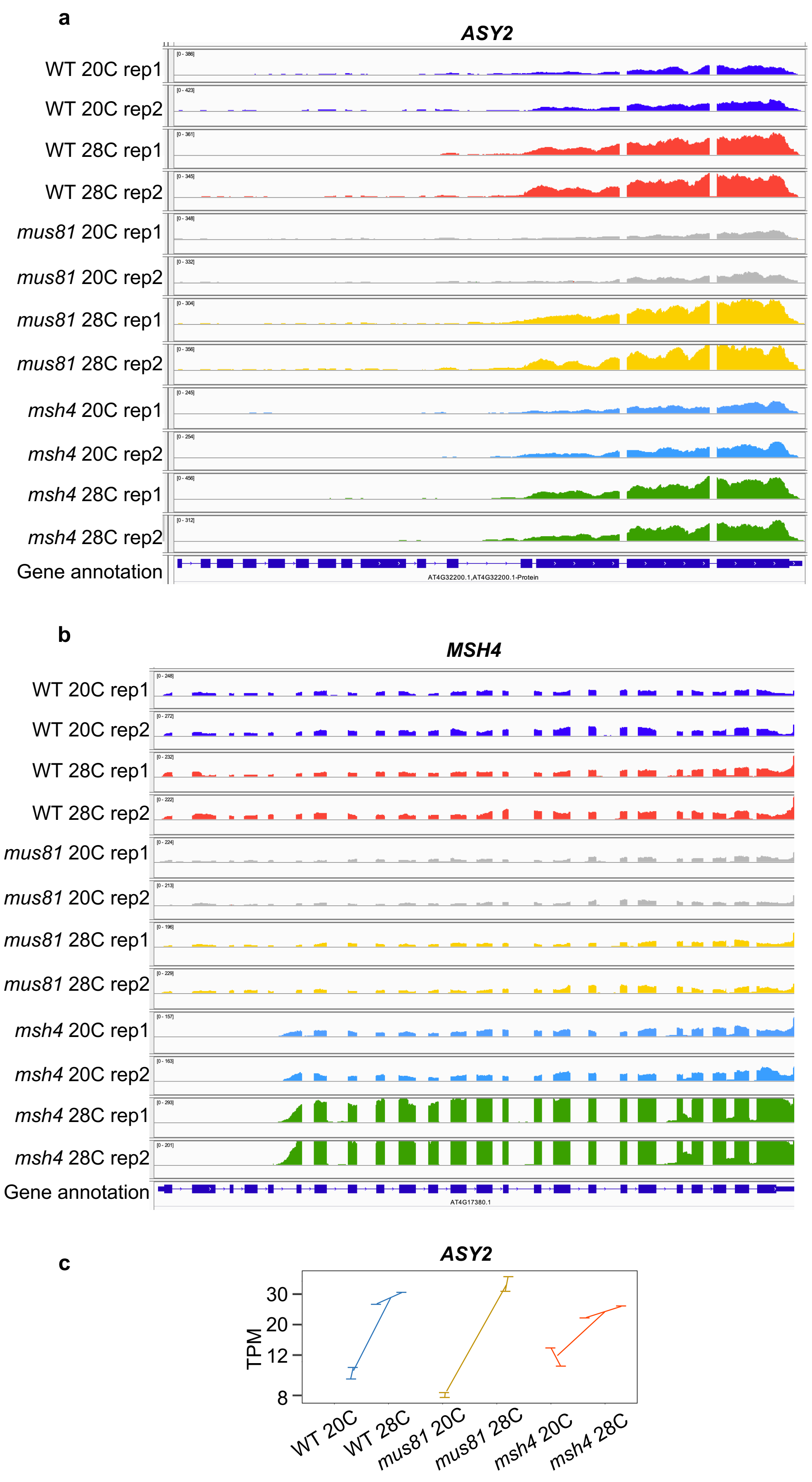

Supplementary Figure 3 Gene expression of *ASY2* and *MSH4* under heat stress in WT, *mus81* and *msh4*.

a Snapshot showing that *ASY2* is an up-regulated DEG in WT and *mus81* but not in *msh4*.

b Snapshot showing atypical up-regulated *MSH4* in *msh4* but not in WT and *mus81*.

c Expression of ASY2 from WT, *mus81* and *msh4* grown under 20°C and 28°C conditions.
