# Supplementary material for: Comparative transcriptomic analysis of thermally stressed Arabidopsis thaliana meiotic recombination mutants
Source: BMC Genomics. 2021 Mar 12;22:181. doi: 10.1186/s12864-021-07497-2 (PMC7953577; doi:10.1186/s12864-021-07497-2)
Supplement: Supplementary file 7 — Additional file 7 : Supplementary Figure 4. A-rich motif enriched in the promoter sequences of 51 randomly selected genes from Arabidopsis Araport11 annotation set. [file 12864_2021_7497_MOESM7_ESM.pdf]

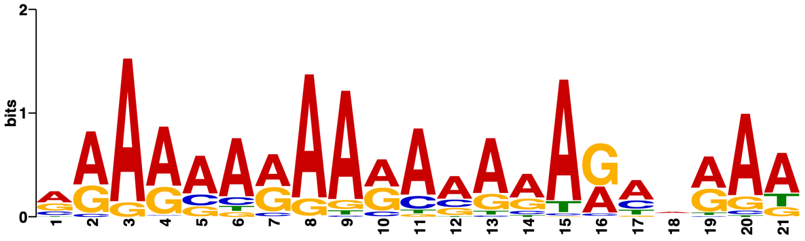

Supplementary Figure 4 A-rich motif enriched in the promoter sequences of 51 randomly selected genes from *Arabidopsis* Araport11 annotation set.
